# Direct and Indirect Haptic Calibration of Visual Size Judgments

**DOI:** 10.1371/journal.pone.0025599

**Published:** 2011-10-13

**Authors:** Monica Gori, Alessandra Sciutti, David Burr, Giulio Sandini

**Affiliations:** 1 Robotics, Brain and Cognitive Sciences Department, Istituto Italiano di Tecnologia, Genova, Italy; 2 Department of Psychology, University of Florence, Florence, Italy; 3 Istituto di Neuroscienze del CNR, Pisa, Italy; McMaster University, Canada

## Abstract

It has long been suspected that touch plays a fundamental role in the calibration of visual perception, and much recent evidence supports this idea. However, as the haptic exploration workspace is limited by the kinematics of the body, the contribution of haptic information to the calibration process should occur only within the region of the haptic workspace reachable by a limb (peripersonal space). To test this hypothesis we evaluated visual size perception and showed that it is indeed more accurate inside the peripersonal space. We then show that allowing subjects to touch the (unseen) stimulus after observation restores accurate size perception; the accuracy persists for some time, implying that calibration has occurred. Finally, we show that observing an actor grasp the object also produces accurate (and lasting) size perception, suggesting that the calibration can also occur indirectly by observing goal-directed actions, implicating the involvement of the “mirror system”.

## Introduction

All measurement systems require calibration to become and remain accurate. Three hundred years ago, in his famous *essay towards a new theory of vision*, Bishop George Berkeley pointed out that vision has no direct access to attributes such as distance, solidity and “bigness” [1]. These must be learned, he claimed, by association with the more direct *tangible* information from touch. This claim has often been paraphrased as “touch educates vision”, which we could usefully rephrase as “touch *calibrates* vision”. From observations of a man recovering from blindness, Richard Gregory concluded that “Vision depends on knowledge derived from active exploration and is limited to the space of tactile experience” [2], [3]. Indeed, since vision is often distorted [4], [5], [6] the haptic feedback may be fundamental to improve visual perception through calibration. Calibration usually refers to the process of adaptation as a way of producing environmentally geared behavior [7]. It has been a central topic for many years and its role has been widely investigated in relation with vision and haptics [e.g. 7], [8], [9], [10], [11], [12], [13]. In particular, calibration has been defined as the use of an error signal (e.g. visual or haptic) to refine performance [9]. Many studies have investigated the role of short term adaptation (calibration) in adults showing a strong plasticity [8], [14], [15], [16], [17], [18].

However, calibration may also be fundamental to more gradual processes occurring during development, in which a sensory modality calibrates (or teaches) the others about some properties of the world (*developmental* calibration). For instance, recent studies reinforce the suggestion that the haptic system has a role in calibration of the visual system in judgments of size during development [19], [20]. In children younger than 8 years, haptic information dominates vision in size judgments, even though haptic judgments are less precise [19]. However, for orientation judgments, vision dominates touch, suggesting that for that attribute vision is the calibrator. Indeed, children born without sight show impaired haptic orientation discrimination compared with controls, but as good or better size discrimination [20].

The different sensory modalities operate over different spatial ranges. For example vision operates over distances as large as kilometers, while the haptic system is limited by the shape and size of the body. If touch is indeed important for calibrating vision during development, particularly for size discrimination this *developmental* calibration should be limited to the region in space reachable by the hands (peripersonal space), a radius of about 60 cm in adults. We tested this hypothesis in this study by measuring size discriminations inside and outside the haptic workspace, and demonstrated systematic biases outside the peripersonal space. We then went on to show that the biases could be reduced or eliminated by allowing subjects to touch the object after it has been observed when out of reach, that is allowing for a calibration mediated by a direct haptic feedback. Finally we show that observing an actor grasping the object in the far space also reduces considerably the errors in size estimation.

## Materials and Methods

**Figure 1:**
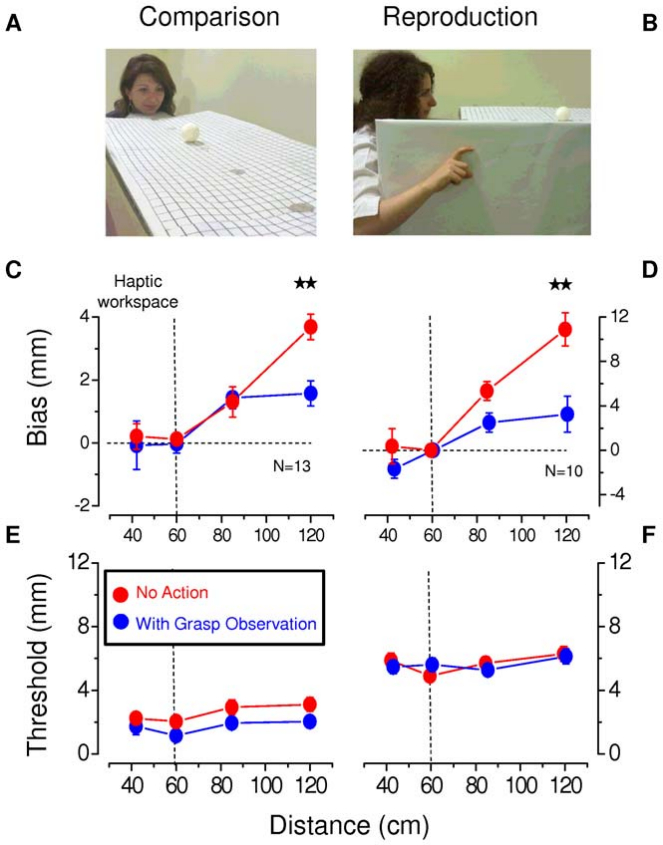
Setup and results for size judgment tasks. **A.** Illustration of the *comparison task*. **B.** Illustration of the *reproduction task*. **C.** Mean bias in size perception (13 subjects) as a function of sphere position for the *comparison task*. The red line and dots refer to the average responses when only the sphere was presented (“no-action” condition). The blue line and dots refer to the average response when subjects observed a grasping action (“grasp- observation” condition). The vertical dashed line indicates the limit of the haptic workspace: The 42.5 and the 60 cm distances fall within the haptic workspace while the 85 and 120 cm distances fall beyond it. Error bars represent ±1 SEM of inter-subject variability. **D.** Same as C for the *reproduction task* (10 subjects). **E.** Average precision thresholds as a function of sphere position for the *comparison task*. The thresholds are an indication of consistency of the response, given by the standard deviation of the cumulative Gaussian psychometric function that fits the comparison data. Error bars represent ±1 SEM of inter-subject variability (same conventions as Fig. 1C). **F.** Same as E for the *reproduction task*. Here the thresholds are the standard deviations of the distributions of matched sizes. In all cases, two stars represent a significance level of less than 0.01 and one star a significance level of less than 0.05 in a repeated measures two-way ANOVA, and Bonferroni post-hoc test.

The stimuli for this study were real physical spheres of about 5 cm diameter, produced on a 3D printer. The spheres were positioned on a plane patterned with a black grid on a white background (see Fig. 1A and 1B and online movie S1). Subjects observed the scene from a fixed height (about 11 cm above the plane), with chin resting on a chin rest fixed at the level of the plane.

For the *comparison task* (first part of the online movie S1), two stimuli were shown successively, the *standard* stimulus with fixed diameter 50 mm (randomly first or second presentation) positioned at 60 cm from the observer, and a *comparison* sphere (varying in diameter from 38 to 62 mm in steps of 1 mm) positioned randomly at one of four chosen distances (42.5 cm, 60 cm, 85 cm and 120 cm). Subjects (7 males and 6 females, average age 29±1) were asked to report which sphere appeared larger. The size of the comparison sphere varied depending on the subject's response, following the QUEST [21] algorithm. 100 trials were collected for each condition. The data were fitted offline with a Cumulative Gaussian function to estimate both PSE (point of subjective equality, given by the mean) and threshold (standard deviation). The bias was computed as the difference between the standard dimension (50 mm) and the estimated PSE. A positive bias indicated size overestimation while a negative number indicated size underestimation. Individual errors were estimated with bootstrap [22].

In separate sessions we measured apparent size by a *reproduction task* (second part of the online movie S1) where subjects (3 males and 2 females of the previous group and a further 2 males and 3 females: average age 28±2) indicated the apparent diameter of a sphere (with a diameter of 42 or 50 mm) by opening their index and thumb. The sphere was positioned at the same four possible distances indicated above. Vision of the hand was occluded. Each condition (10 in total: 2 sizes×5 distances) was repeated 10 times. The aperture between the two fingers was recorded by infrared markers (Optotrak Certus System, Northern Digital Inc., Canada). At the end of each experimental session, the Optotrak markers were calibrated to actual grip span by recording their position while the subject blindly held the spheres. Subjects were recruited from the local university and were tested for about 15 hours if involved in both tests and for about 7 hours for just one test. Subjects started with the index finger touching the thumb, and then adjusted their grip to indicate the apparent size. The aperture was estimated averaging the signal over about half a second after the fingers had reached their final aperture. All measured apertures were normalized by dividing them by the average aperture realized when the sphere was presented at the 60 cm distance (the position of the standard stimulus in the previous task), thus obtaining a dimensionless number. The difference between this ratio and 1 was then multiplied by the real ball dimension, to compute the bias measured as the difference in mm between reproduced and real ball dimension. A positive number indicated overestimation of ball size.

The precision threshold was computed as the standard deviation of the distribution of the estimates. For those subjects tested in both the *comparison* and the *reproduction* tasks, the order of the two tasks was randomly chosen.

We studied four different conditions, illustrated in the on-line movie (Movie S1). In the “no-action” condition subjects were presented visually with spheres positioned at different distances (first part of both *comparison* and *reproduction* movie S1 sections). In the “grasp-observation” condition (second part of both *comparison* and *reproduction* movie S1 sections), subjects observed the same visual scene while the experimenter performed a grasping action on the sphere (or on both the standard and the comparison spheres in the *comparison* task). The “static” condition was identical to the “grasp-observation” condition except that subjects were shown the demonstrator's static hand holding the spheres. In the “backward” condition (the last part of both *comparison* and *reproduction* movie S1 sections) they observed the release of the spheres: the demonstrator initiated the action by touching the sphere in place, then left removing his or her hand (the opposite of grasping). In all conditions the visual scene was maintained identical to the “no-action” condition. We also introduced a “haptic-feedback” condition, identical to the “no-action” condition except that subjects blindly touched the balls after having observed them outside the haptic workspace. This condition was conducted only with the *reproduction task* and only for the 120 cm sphere distance.

All participants gave written informed consent prior to testing. The study was approved by the local ethics committee (Azienda Sanitaria Locale Genovese N.3).

## Results

We used two psychophysical tasks, *comparison* and *reproduction*, to measure visual size perception of objects inside and outside the haptic workspace (“no-action” condition). This task requires that the subject has good *size constancy*, the capacity to scale the retinal image with distance to recover the physical size of the object. The *comparison task* (Fig. 1A) was a two-interval forced-choice procedure, where subjects chose the larger of two spheres, one a 50 mm diameter *standard* positioned at 60 cm from the observer and the other of variable diameter (38–62 mm) randomly placed at one of four chosen distances (42.5 cm, 60 cm, 85 cm and 120 cm). In the *reproduction task* (Fig. 1B) subjects indicated the apparent diameter of a sphere by opening the index and thumb of their hand (occluded from vision).

**Figure 2:**
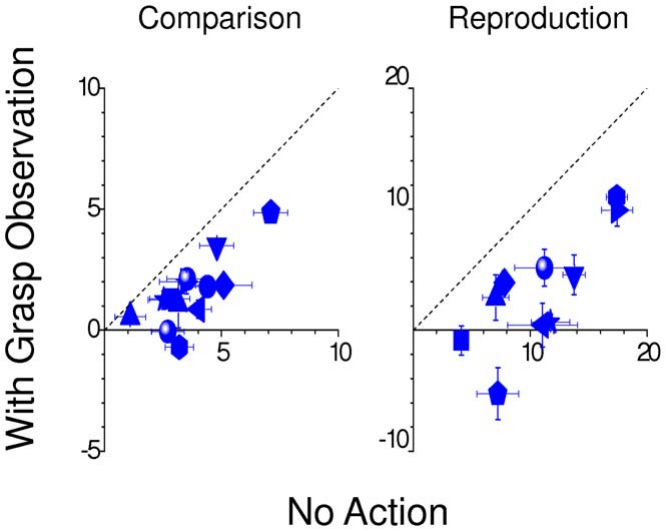
Individual data for “no-action” and “grasp-observation” conditions. Data of individual subjects, plotting the bias in the “grasp-observation” condition against that in the “no-action” condition, for the *comparison task* (A) and *reproduction task* (B) for 120 cm of distance. The error bars show ±1 SEM, estimated by bootstrap for the *comparison task* and from the matching variance for the *reproduction task*.

The mean size estimates for the two tasks are shown by the red symbols in Fig. 1C&D (the blue symbols and lines refer to the second part of the experiment, discussed later). For both tasks, size estimation was unbiased at 42 and 60 cm (constant inside the haptic workspace), but showed a clear overestimation at larger distances, increasing with distance. This means that if two balls of the same size are presented at different distances, the one positioned further away appears larger, even though its retinal image is smaller. This finding suggests that size overestimation, reported for observation of distant objects [e.g. 4], [23], [24], also occurs in adults for nearer objects, provided they are outside the haptic workspace. The bias was systematically observed in all subjects (see Fig. 2), but smaller for the size *comparison* than *reproduction*. Fig. 1E&F shows the precision thresholds (a measure of internal consistency of judgments) for the two tasks. Thresholds were lower for the *comparison task* than for the *reproduction task*, possibly because of noise associated with the reproduction.

**Figure 3:**
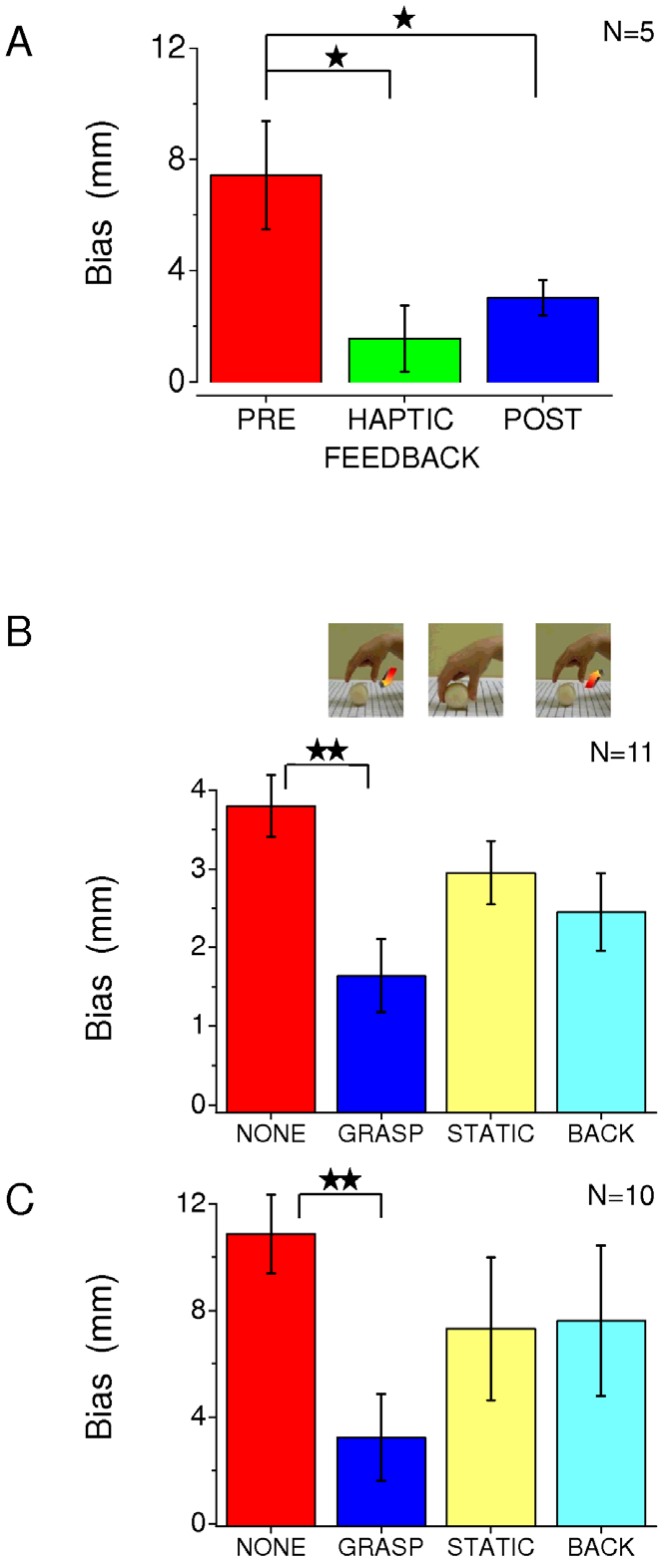
Effect of touch and observation of touch on visual bias. **A.** Average biases (*reproduction task*) of 5 subjects in judging the size of a sphere at 120 cm for the ”no-action” condition (red bar) and when subjects also blindly felt the sphere in the “haptic-feedback” condition (green bar). The blue bar shows the biases of the same subjects retested in the “no-action” task after the “haptic-feedback” test. Error bars represent ±1 SEM of inter-subject variability. **B.** Average biases (*comparison task*) in judging the size perception of a sphere at a distance of 120 cm. The red bar refers to the “no-action” condition, the blue to visual judgments after observing an actor grasping the sphere (“grasp-observation” condition). The yellow and light-blue bars show two control conditions, respectively observation of a stationary hand holding the spheres (“static” condition) and observing a releasing movement (“backward” condition). Error bars represent ±1 SEM of inter-subject variability. **C.** Same as B for *reproduction task*. In all cases, one and two stars represent, respectively, significance levels of less than 0.05 and 0.01 in a repeated measures one-way ANOVA and Bonferroni post-hoc test.

To test whether the systematic bias in size perception in far-space reflects lack of haptic calibration, we repeated the measurements while allowing subjects to touch the ball after having observed it outside the haptic workspace (“haptic-feedback” condition). Fig. 3A shows the results for the reproduction condition, for the ball at 120 cm. Without haptic feedback, we observed an average bias of about +8 mm (red bar, Fig. 3A) and when observers were allowed to touch the ball (without seeing it) this bias was reduced considerably to about +2 mm (green bar, Fig. 3A). Interestingly, when subjects were retested in the “no-action” condition, after having been tested in the “haptic-feedback” condition, the judgments remained unbiased (blue bar, Fig. 3A). These results suggest that the haptic sense can calibrate vision, and that the calibration persists for some time afterwards.

Encouraged by these results, we asked whether it was necessary for the subject to actually touch the ball, or whether another person could act as a “proxy” for this action: could observing the ball being grasped by an actor calibrate visual size? In this experiment, the subject observed the ball, followed by the experimenter grasping it (“grasp-observation” condition, see Materials and Methods and online movie S1). The results are shown by the blue symbols of Fig. 1C&D. Observing the grasping action of the experimenter greatly reduced the overestimation for both the *comparison* and *reproduction* tasks outside the haptic workspace. The reduction in bias was large and robust, particularly at 120 cm where the improvement in accuracy was highly significant for both tasks. A repeated-measures two-way analysis of variance (ANOVA) was conducted for each task (*comparison* and *reproduction*) considering as factors the experimental condition (two levels: no-action and grasp-observation) and the distance (four levels: 42.5, 60, 85, 120 cm). For both tasks a significant effect of each independent factor and their interaction (p<0.01) was found. In particular a Bonferroni post hoc test on the interaction individuated as significant the difference between the biases for the “no-action” and the “grasp-observation” conditions at 120 cm distance.

Figure 2 shows individual data at this distance for the two tasks: without exception, every subject showed an improvement in accuracy when observing the grasping action (all points below the equality line). Not only did observation of the grasping action reduce the bias: for the *comparison task* it also improved precision (blue symbols of Fig. 1E&F, p<0.01 of the experimental condition factor, in a repeated measures two-way ANOVA on the SDs with the same structure described above for the PSEs). No improvement (nor any significant difference) was observed for the *reproduction task*, possibly because the extra noise involved in making the reproduction swamped the sensory noise, present in both the “grasp-observation” and “no-action” conditions.

The grasping action of the demonstrator introduces many additional cues that may have aided size discrimination. For example, as the actor was always the same person in a given session, the hand size could have acted as a reference for size discrimination. Similarly, the movement of the hand could in some way help to make a more accurate judgment of distance, which could aid size constancy. We therefore ran two separate control conditions (see online movie S1 for further details). In one, the hand was seen stationary on the ball (“static” condition), lightly holding the ball between thumb and index finger, at the same angle as the “grasp-observation” condition. This did not significantly reduce bias (yellow bar of Figs. 3B&C, p>0.05, pair-sample t-test). In a second control the subjects saw the grasping action reversed in time: on eye-opening the actor's hand released the sphere and withdrew with a movement that mirrored the grasping action (“backward” condition). Again this did not reduce significantly the bias (light blue bar Figs. 3B&C, p>0.05, pair-sample t-test). The only significant difference (repeated measures one-way ANOVA and Bonferroni post-hoc test) of all the possible comparisons in both measurement conditions was between the vision only condition and the natural forward grasping (red and blue bars of Figs. 3B&C). Static and backward conditions are thus in between the “no-action” and the “grasp-observation” conditions.

## Discussion

Although most recent work on multi-sensory interactions has concentrated on sensory *fusion*, the efficient combination of information from all the senses, an equally important potential function is *calibration*. The results of this study reinforce previous findings in suggesting an important role for the haptic system in calibrating vision [7], [9], [17]. Visual judgments of size outside the haptic workspace are biased in adult subjects, but after allowing the subjects to touch the physical ball the bias is greatly reduced, and this improvement persists even when subjects no longer touch the ball, implying a recalibration of the system.

A further result of our study was that observing the goal-directed grasping action of another individual had a similar effect of bias reduction to directly touching the ball. Control conditions, such as backward grasping and observation of a stationary hand did not reduce the bias. What information is then used for the calibration? Presumably visual information, such as the size of the hand, or motion cues, to judge depth more accurately, contribute to achieve size-constancy. Importantly, however, this information on its own is not sufficient, as we show in the two control conditions. It seems that for this information to be used for calibration, it must be linked to a goal-directed action. During “forward” grasping, the grip aperture unfolds predictably, scaling to the size of the object as the hand approaches it (grip size scaling) [25], [26], [27] and this would be valuable information about object size from viewing the approach as well as the moment of contact. A possible candidate for this process could be the mirror neuron system [28]. These neurons, originally discovered in the ventral premotor area F5 of the macaque monkey [29], respond both during action execution and observation and are thought to constitute a system fundamental for understanding goal-directed actions [for recent review see 30], [31].

An important path to calibration of visual size is through the haptic system, both in adults and children. However, when objects are out of reach, direct haptic feedback is not available. The observation of someone else grasping the same object could provide a compensation for the missing haptic feedback through mirror system activation. Grasping observation could produce motor activation analogous to that available to the observer during grasp execution. There are many circumstances in which individuals are unaware of visual cues, but are able to use them when action execution is required [32], [33], and motor activation could be responsible for this process. In our case visual cues such as hand movements or hand size, which apparently are not useful by themselves during visual size estimation, could become available to the perceptual system when there is a motor activation derived by the mirror neuron system. The mirror system seems to be a plausible candidate to activate this cross-modal calibration. We therefore suggest that an additional function for the mirror neuron system may be to extend haptic calibration of vision outside peripersonal space, allowing extra visual information (such as hand size) to act through the neural circuitry that normally subserves direct haptic calibration of vision. Interestingly, recent evidence has shown that in macaque monkeys, different populations of mirror neurons respond to action observed inside and outside the haptic workspace [34]. The ability of understanding the correct size of objects outside our action range could be especially useful during interaction with other people, to optimize collaborative use of the object at hand.

## Supporting Information

Movie S1 **The movie shows the** ***comparison*** **(first section) and** ***reproduction*** **(second section) tasks for the “no-action” (first part of both sections), “grasp-observation” (second part of both sections) and “backward” (last part of both sections) conditions.** In the video the chin rest has been removed for clarity. In the last section of the movie a detail of the hand motion for both “grasp- observation” and “backward” conditions is shown.
(WMV)
